# Synovial DKK1 expression is regulated by local glucocorticoid metabolism in inflammatory arthritis

**DOI:** 10.1186/ar4065

**Published:** 2012-10-18

**Authors:** Rowan Hardy, Maria Juarez, Amy Naylor, Jinwen Tu, Elizabeth H Rabbitt, Andrew Filer, Paul M Stewart, Christopher D Buckley, Karim Raza, Mark S Cooper

**Affiliations:** 1 Centre for Endocrinology, Diabetes and Metabolism, University of Birmingham, Queen Elizabeth Hospital, Edgbaston, Birmingham, B15 2TH, UK; 2 Rheumatology Research Group, MRC Centre for Immune Regulation, The Institute of Biomedical Research, University of Birmingham, Birmingham, B15 2TH, UK; 3 Bone Research Program, ANZAC Research Institute, 1A Hospital Road, Concord, Sydney NSW 2139, Australia; 4 Department of Rheumatology, Sandwell and West Birmingham Hospitals NHS Trust, City Hospital, Dudley Road, Birmingham, B18 7QH, UK

## Abstract

**Introduction:**

Inflammatory arthritis is associated with increased bone resorption and suppressed bone formation. The Wnt antagonist dickkopf-1 (DKK1) is secreted by synovial fibroblasts in response to inflammation and this protein has been proposed to be a master regulator of bone remodelling in inflammatory arthritis. Local glucocorticoid production is also significantly increased during joint inflammation. Therefore, we investigated how locally derived glucocorticoids and inflammatory cytokines regulate DKK1 synthesis in synovial fibroblasts during inflammatory arthritis.

**Methods:**

We examined expression and regulation of DKK1 in primary cultures of human synovial fibroblasts isolated from patients with inflammatory arthritis. The effect of TNFα, IL-1β and glucocorticoids on DKK1 mRNA and protein expression was examined by real-time PCR and ELISA. The ability of inflammatory cytokine-induced expression of the glucocorticoid-activating enzyme 11beta-hydroxysteroid dehydrogenase type 1 (11β-HSD1) to sensitise fibroblasts to endogenous glucocorticoids was explored. Global expression of Wnt signalling and target genes in response to TNFα and glucocorticoids was assessed using a custom array.

**Results:**

DKK1 expression in human synovial fibroblasts was directly regulated by glucocorticoids but not proinflammatory cytokines. Glucocorticoids, but not TNFα, regulated expression of multiple Wnt agonists and antagonists in favour of inhibition of Wnt signalling. However, TNFα and IL-1β indirectly stimulated DKK1 production through increased expression of 11β-HSD1.

**Conclusions:**

These results demonstrate that in rheumatoid arthritis synovial fibroblasts, DKK1 expression is directly regulated by glucocorticoids rather than TNFα. Consequently, the links between synovial inflammation, altered Wnt signalling and bone remodelling are not direct but are dependent on local activation of endogenous glucocorticoids.

## Introduction

During adult life, bone formation and resorption are normally tightly coupled such that the amount of bone formed is similar to the amount resorbed. However, in patients with chronic inflammatory arthritis (for example, rheumatoid arthritis (RA)) bone remodelling is abnormal [1,2]. Bone resorption is increased due to increased activity of osteoclasts whereas bone formation by osteoblasts is suppressed. The uncoupling of formation from resorption results in bone loss and an increased risk of fractures [3]. A similar process is seen in states of systemic glucocorticoid excess such as Cushing's syndrome or during treatment with therapeutic glucocorticoids, but circulating glucocorticoid levels in patients with RA are not elevated [4]. We have previously hypothesised that the bone loss seen in inflammatory arthritis is secondary to local glucocorticoid activation through the 11beta-hydroxysteroid dehydrogenase type 1 (11β-HSD1) enzyme [5]. This enzyme converts inactive steroids (such as cortisone and prednisone) to their active counterparts (cortisol and prednisolone) [6,7]. Patients lacking this enzyme are unresponsive to cortisone acetate or prednisone therapy due to their inability to activate these steroids *in vivo *[8]. We have previously demonstrated that this enzyme is highly expressed in human primary synovial fibroblasts and synovial tissue explants [9,10]. *In vitro*, the expression and activity of this enzyme increase dramatically in these cells and tissues in response to TNFα or IL-1β [9-11]. In patients with RA, 11β-HSD1 activity in synovial tissue and total body measures of 11β-HSD1 activity are increased and correlate with serum markers of inflammation [10]. In a rodent model of inflammatory arthritis, 11β-HSD1 activity and expression in the joint are increased, and activity is reduced by anti-TNF therapy [12]. Thus the level of active glucocorticoids within the joint, and specifically within synovial fibroblasts, appears to be high during inflammatory arthritis.

Recently, secretion of the Wnt antagonist dickkopf-1 (DKK1) has been proposed to be a master regulator of bone remodelling in inflammatory arthritis [13]. DKK1 suppresses osteoblast differentiation but also decreases the expression of osteoprotegerin (OPG) leading to increased osteoclastogenesis. DKK1 is synthesised by murine synovial fibroblasts in response to inflammation through a TNFα-dependent mechanism [13]. Neutralisation of DKK1 in mice using anti-DKK1 antibodies reversed the bone loss seen in inflammatory arthritis and resulted in the formation of new bone near the areas of greatest inflammation. In osteoblasts, mesenchymal cells that are developmentally closely related to synovial fibroblasts, glucocorticoids are a very powerful inducer of DKK1 and this effect has been proposed as the mechanism that mediates bone loss due to systemic glucocorticoid excess [14]. Given the increase in 11β-HSD1 expression that we have observed in synovial fibroblasts, we hypothesised that the regulation of synovial fibroblast DKK1 expression by inflammation was indirect and dependent on the local generation of glucocorticoids within the synovial fibroblasts. Therefore, we assessed the regulation and relative expression of DKK1 following treatments with both glucocorticoids and inflammatory cytokines in primary synovial fibroblasts.

## Materials and methods

### Patients

Biopsies of matched synovium and skin were obtained during hip, knee or elbow arthroplasty from patients with RA (based on the 1987 American College of Rheumatology (formally the American Rheumatism Association) criteria), osteoarthritis (OA) and ankylosing spondylitis (AS) (based on the modified New York criteria). Tissue was taken on ice from the operating theatre and synovial tissue was prepared within 2 hours by removing any adherent non-synovial tissue. Tissue explant experiments were performed on 20 mg sections prior to enzyme assay or ELISA. Skin tissue was prepared by removing the subcutaneous fat and dividing into 20 mg pieces prior to enzyme assay or ELISA. Primary cultures of synovial and dermal fibroblasts were generated as described previously [9]. Fibroblasts were treated with 0.01 to 10 ng/ml TNFα (R&D Systems, Abingdon, UK) or 0.1 to 100 nmol/l of dexamethasone (DEX), cortisol or cortisone with or without 1 μmol/l of the 11β-HSD inhibitor glycyrrhetinic acid (GE) for 24 hours before harvesting for mRNA analysis or for 48 hours before measuring DKK1 in culture media. All studies had ethical approval from the Local Ethics Committee and informed consent was obtained prior to taking of samples.

### RNA extraction and reverse transcription

RNA was extracted from cultured fibroblasts using the single-step extraction method (TRI Reagent, Sigma-Aldrich, Poole, UK). Briefly, confluent monolayers of synovial fibroblasts in 6-well plates were lysed in 1 ml of TRI Reagent and RNA isolated as per the manufacturers protocol. RNA were then reverse transcribed using random hexamers in a 20 μl volume, as stated in the manufacturer's protocol (Promega, Madison, WI, USA) [15].

### Real-time PCR

Probes and primers were based on Assay-on-Demand™ sequences (Applied Biosystems, Warrington, UK). mRNA levels for DKK1 (Hs00183740_m1), DKK2 (Hs00997455_m1), WNT2 (Hs00608224_m1) and FRZB (Hs00173503_m1) were assessed using real-time PCR in an ABI 7500 system (Applied Biosystems, Warrington, UK) using a previously reported technique [11]. Reactions contained TaqMan universal PCR master mix (Applied Biosystems, Warrington, UK), 900 nmol primers, 100 to 200 nmol TaqMan probe and 50 ng cDNA. Primers for 18S (Hs03928985_g1) were used as an internal reference. All target gene probes were labelled with the fluorescent label FAM, and the 18S probe with the fluorescent label VIC. Reactions occurred as follows: 50°C for 2 minutes, 95°C for 10 minutes, 40 cycles of 95°C for 15 seconds and 60°C for 1 minute. Data were obtained as Ct values (the cycle number at which logarithmic PCR plots cross a calculated threshold line) and used to determine ΔCt values (Ct of target gene - Ct of housekeeping gene) as raw data for gene expression (high ΔCt = low gene expression). The fold change in gene expression was determined by subtracting ΔCt values for treated cells from their respective control samples. The resulting ΔΔCt values were then used to calculate fold change in gene expression according to the equation 2^-ΔΔCt^.

### Real-time PCR custom TaqMan gene array

Global expression of Wnt signalling and target genes was assessed using the predefined Wnt signalling gene format with TaqMan Express Plates (Catalogue number 4391524, Applied Biosystems, Warrington, UK). Both 18S and GAPDH were used as an internal reference. FAM-labelled primers and probes were reconstituted to 20 μl in TaqMan universal PCR master mix (Applied Biosystems, Warrington, UK) containing 50 ng of target cDNA. Real-time PCR was performed using an ABI 7500 system (Applied Biosystems, Warrington, UK) using a previously reported technique [11]. Reaction conditions and data analysis were performed as reported for real-time PCR. Array data were submitted to the Gene Expression Omnibus (GEO) repository and given the designation GSE37520.

### Analysis of DKK1 levels by ELISA

DKK1 levels in supernatants from cultured cells were measured using a commercially available sandwich ELISA (Catalogue Number: DY1906, R&D systems, Abingdon, UK). Briefly, synovial fibroblasts were seeded at 100,000 cells per well in a 6-well plate. Media was conditioned for 48 hours in the presence or absence of appropriate treatments. Conditioned media was collected and stored at -80°C prior to analysis in triplicate by ELISA. Data were expressed as ng/ml of DKK1 in culture media after 48 hour culture with 100,000 cells from a minimum of three separate fibroblast lines.

### Statistics

Data were reported as the mean ± standard deviation (SD) of synovial explants from separate individuals (unless otherwise stated). Multiple comparisons were assessed using one-way ANOVA with a Dunnett's post hoc analysis. Regression analysis and students paired *t *tests were performed using SPSS Data Editor (SPSS Inc., Chicago, IL, USA).

## Results

### Induction of DKK1 expression in human primary synovial fibroblasts is by glucocorticoids and not TNFα/IL-1β

**Figure 1:**
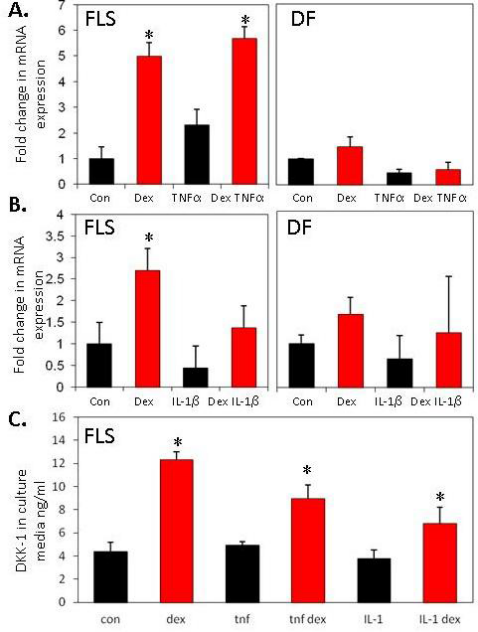
**Effects of glucocorticoids/proinflammatory cytokines on DKK1 expression in primary synovial fibroblasts (FLS) or dermal fibroblasts (DF)**. Results shown are the combined duplicates of four separate FLS or DF cell-lines. **(A) **Effect of dexamethasone (100 nM), TNFα (10 ng/ml), or **(B) **IL-1β (10 ng/ml) on DKK1 mRNA expression in FLS. **(C) **Effect of dexamethasone (100 nM), TNFα (10 ng/ml) and IL-1β (10 ng/ml) on secretion of DKK1 protein in FLS. Dexamethasone but not TNFα or IL-1β, induces significant secretion of DKK1 protein from FLS. *P < 0.05, **P < 0.01.

The effects of glucocorticoids and proinflammatory cytokines on DKK1 expression were examined in primary human synovial fibroblasts. Dexamethasone (100 nM) caused a substantial and statistically significant increase in DKK1 mRNA expression (4.9-fold vs control; *P *< 0.05) (Figure 1A). TNFα (10 ng/ml) caused a small and non-significant increase in DKK1 mRNA expression (2.3-fold vs control; NS). In this experimental set-up, levels of endogenous glucocorticoids in the media were below those required to allow an indirect glucocorticoid-mediated effect of TNFα expression on DKK1 mRNA expression. TNFα did not further augment the effect of glucocorticoid treatment on DKK1 mRNA expression when the two were combined. Similar results were obtained when the effect of dexamethasone on DKK1 mRNA expression was compared with IL-1β (10 ng/mL) (Figure 1B) with IL-1β having no direct effect on DKK1 mRNA expression. There was no effect of dexamethasone, TNFα or IL-1β treatment on DKK1 mRNA expression in dermal fibroblasts that were used as non-synovial, fibroblast controls. Dexamethasone also caused a significant increase in DKK1 protein secretion in synovial fibroblast cultures but TNFα and IL-1β had no overall effect (control, 4.4 ± 0.4 vs DEX, 12.3 ± 0.4 ng/ml; *P *< 0.05) (Figure 1C).

**Figure 2:**
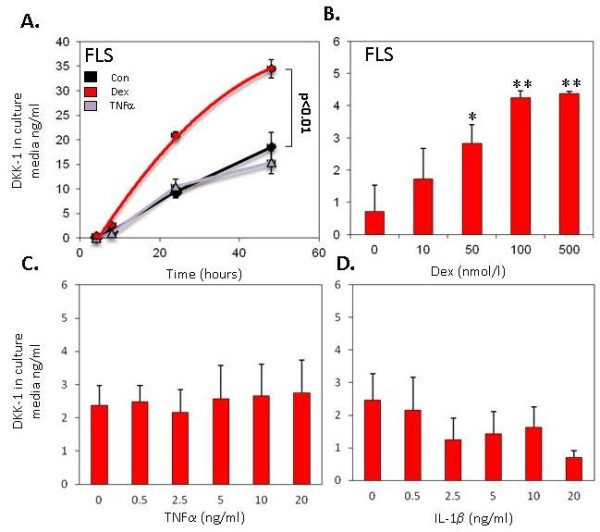
**Regulation of DKK1 expression by glucocorticoids**. **(A) **Time course for induction of DKK1 protein expression by synovial fibroblasts (FLS) in response to dexamethasone or TNFα. **(B) **Dose response curves for the secretion of DKK1 in response to dexamethasone (expressed relative to baseline expression). *P < 0.05, **P < 0.01 relative to control.

The time course of DKK1 secretion in response to dexamethasone is shown in Figure 2A. DKK-1 expression increased in a time-dependent manner. There was no increase in DKK1 secretion with TNFα above basal expression at any time point, whilst dexamethasone significantly increased expression at all time points examined (48 hour control, 18.6 ± 2.8 ng/ml vs 48 hour DEX, 34.5 ± 1.8 ng/ml; *P *< 0.05). The expression of DKK1 in response to dexamethasone was dose dependent and reached a peak at 100 nM dexamethasone (control, 1 ± 1.1 ng/ml vs DEX 100 nmol/l, 6.0 ± 1.4 ng/ml; *P *< 0.05) (Figure 2B). In contrast, TNFα and IL-1β at any dose did not significantly increase secreted DKK1 in dose-response experiments (Figure 2C-D).

### Activation of glucocorticoids within synovial fibroblasts regulates DKK1 synthesis

**Figure 3:**
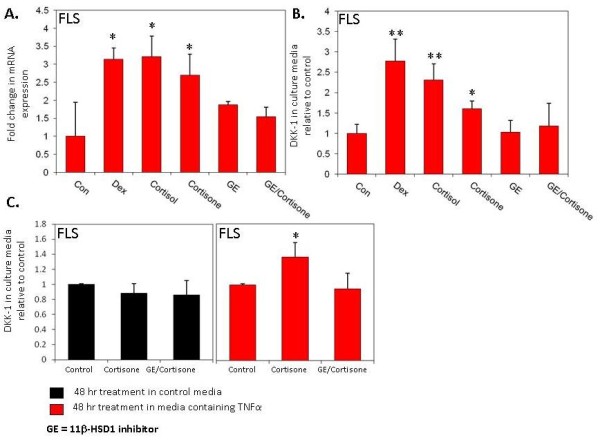
**The effect of endogenous glucocorticoids +/- 11β-hydroxysteroid dehydrogenase (11β-HSD) enzyme inhibitor on DKK1 expression**. **(A) **Effect of various glucocorticoids on DKK1 mRNA expression in synovial fibroblasts (FLS). Cortisone caused a significant increase in DKK1 mRNA, an effect blocked by the 11β-HSD1 inhibitor glycyrrhetinic acid (GE). **(B) **Effect of glucocorticoids on DKK1 protein secretion in FLS and dermal fibroblasts (DF). **(C) **Sensitisation of DKK1 secretion by FLS to the effects of cortisone (E) by pretreatment with TNFα for 48 hours. *P < 0.05, **P < 0.01 relative to control. Results shown are the combined duplicates of four separate FLS lines.

Since dexamethasone is a synthetic glucocorticoid, we further examined whether endogenous glucocorticoids also have the same effect on DKK1 expression (Figure 3). The effect of cortisol was similar to that of dexamethasone in inducing DKK1 mRNA and protein expression (DEX; mRNA 3.1-fold, protein 2.7-fold ± 0.53; cortisol, mRNA 3.2-fold, protein 2.3-fold ± 0.39 vs control; *P *< 0.05) (Figure 3A and 3B). Cortisone was also found to significantly induce DKK1 mRNA and protein expression (cortisone; mRNA 2.7-fold, protein 1.6-fold ± 1.8 vs control; *P *< 0.05). To function effectively as a glucocorticoid receptor agonist, cortisone needs to be converted to cortisol by the 11β-HSD1 enzyme [4]. Inhibition of the 11β-HSD1 enzyme using glycyrrhetinic acid (GE) blocked the effect of cortisone on DKK1 expression. As observed for dexamethasone, neither cortisol nor cortisone had an impact on DKK1 expression in dermal fibroblasts (data not shown). Given the lack of direct induction of DKK1 expression with TNFα, we explored whether TNFα treatment could sensitise synovial fibroblasts to cortisone through induction of 11β-HSD1 activity (Figure 3C). The duration of incubation of synovial fibroblasts with cortisone was reduced to 5 hours, such that conversion of cortisone to cortisol was insufficient to have any effect on DKK1 protein synthesis under basal conditions. Under these conditions, pretreatment with TNFα sensitised synovial fibroblasts to the effects of cortisone. This effect was blocked by an inhibitor of 11β-HSD1 activity, confirming an indirect effect of TNFα via upregulation of 11β-HSD1 activity.

### Comparison between DKK1 synthesis in patients with RA, OA and AS

To assess whether the changes observed were specific to synovial fibroblasts of RA origin, the effect of glucocorticoids on DKK1 protein secretion was examined in synovial fibroblasts isolated from patients with different arthritides (OA and AS, *n *= 5 in total). As with synovial fibroblasts from patients with RA, both cortisol and cortisone were able to induce DKK1 synthesis (data not shown). There was a suggestion that the basal expression level of DKK1 was lower in patients with AS (*P *< 0.05 when AS cells were compared to other synovial fibroblasts) but the number of patients with AS limited the robustness of this finding.

### Glucocorticoids modify Wnt gene expression within synovial fibroblasts in a coordinated manner

**Figure 4:**
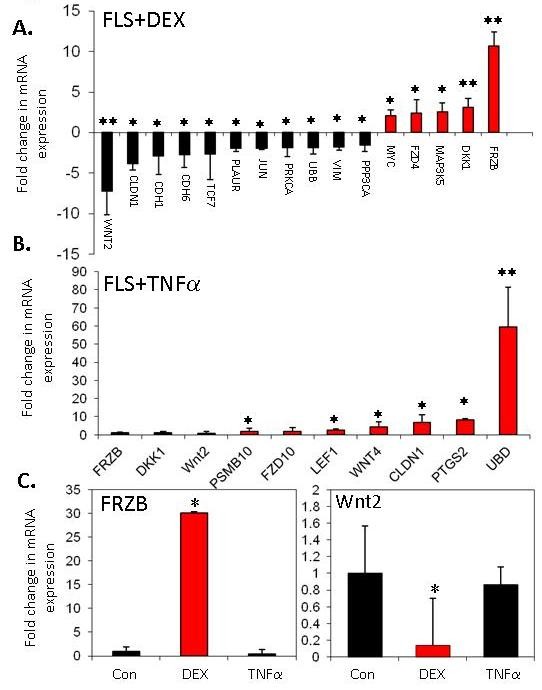
**Effects of glucocorticoids or TNFα on Wnt-related gene expression**. **(A and B) **Effect of glucocorticoid treatment (100 nM dexamethasone) or TNFα (10 ng/ml) on expression of Wnt-related genes (*n *= 96) was examined using a targeted microarray. Genes with the greatest fold change are shown (the effect of TNFα on DKK1 and FRZB expression was not significant). A full list of the genes included in the array and their full gene titles is given in Additional file 1, Table S1. **(C) **Real-time PCR validation of changes in mRNA expression for FRZB and WNT2 in response to dexamethasone (DEX) or TNFα. *P < 0.05, **P < 0.01 relative to control. Results shown are the combined results of three separate FLS lines.

The effect of glucocorticoid treatment (100 nM dexamethasone) or TNFα (10 ng/ml) on expression of a range of Wnt-related genes (*n *= 96) was examined using a targeted microarray (a full list of the genes included in the array is given in Additional file 1, Table S1 and data can be found at the GEO repository with reference number GSE37520). Genes with the greatest fold change are shown in Figure 4. In keeping with the results shown earlier, glucocorticoid treatment resulted in a significant increase in DKK1 expression (3.1-fold vs control; *P *< 0.05). There was, in addition, a substantial induction of the frizzled-related protein-B (FRZB) gene (also known as secreted frizzled related protein-3 (SFRP3)) (10.6-fold vs control; *P *< 0.05). FRZB, like DKK1, is also a secreted inhibitor of Wnt signalling. In keeping with a coordinated effect to reduce Wnt signalling, the expression of WNT2 was substantially reduced (7.2-fold vs control; *P *< 0.05). The effect of TNFα on expression of Wnt-related genes was less dramatic and did not suggest a coordinated effect on the expression of secreted Wnts and their antagonists. The genes with greatest upregulation in response to TNFα treatment were the ubiquitin-like gene UBD and cyclooxygenase-2 (PTGS2) (59.4- and 8.3-fold respectively; *P *< 0.05). TNFα had no significant effect on DKK1 and FRZB expression. Real-time PCR was used to validate the changes in mRNA expression for FRZB and WNT2 in response to dexamethasone and TNFα (Figure 4C).

## Discussion

**Figure 5:**
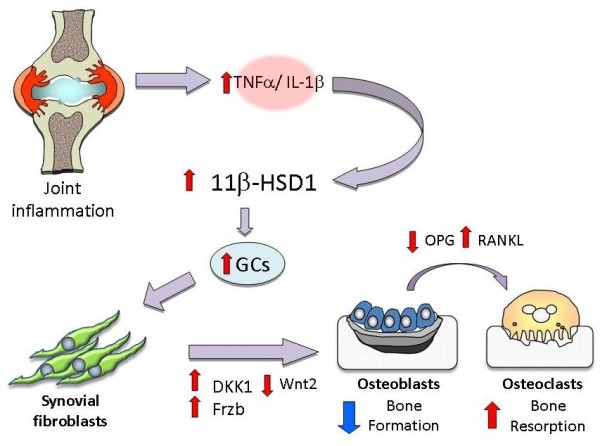
**Role of local glucocorticoid generation in inflammatory changes in bone**. Schematic illustration of the mechanism by which synovial inflammation interacts with local generation of active glucocorticoids to modulate Wnt signalling in osteoblasts.

Bone is an important target tissue in patients with RA, with many patients developing local erosions and periarticular and generalised osteoporosis. Previous studies have highlighted the central importance of synovial fibroblasts in the abnormal bone remodelling associated with joint inflammation [13]. These effects were assumed to be due to direct effects of inflammatory mediators, principally on expression of the Wnt signalling antagonist DKK1. Indeed, a growing body of work has demonstrated that this secreted factor is markedly elevated in the serum of patients with rheumatoid arthritis, where its levels positively correlate with multiple markers of disease activity [13,16]. Most importantly, DKK1 positively correlated with erosive bone loss in patients with rheumatoid arthritis, supporting a central role for this factor in inflammatory bone loss. Our findings demonstrate that the effect of inflammation on DKK1 synthesis is not direct, but instead depends on an increase in local glucocorticoid levels through the induction of the 11β-HSD1 enzyme (illustrated schematically in Figure 5). High levels of glucocorticoids are linked with a range of conditions that detrimentally affect bone through uncoupling bone formation from resorption, but this is the first study to show a link between effects of glucocorticoids on synovial tissue and uncoupling of bone metabolism through a paracrine effect. Glucocorticoids also cause a coordinated change in production of Wnt signalling modulators by synovial fibroblasts that extends beyond DKK1 with an increase in several antagonists and a suppression of agonists. These findings are in accordance with the effects of glucocorticoids on Wnt production by osteoblasts [14], a cell type closely related developmentally to the synovial fibroblast.

It is well established that high circulating levels of endogenous or exogenous glucocorticoids suppress bone formation. This is at least partly through a direct mechanism, since mice with osteoblast-specific deletion of the glucocorticoid receptor alpha (GRα) or overexpression of 11β-HSD2 (which inactivates glucocorticoids) within osteoblasts are protected against the effects of high-dose glucocorticoids on bone [17,18]. These results suggest that the development of synovial hypertrophy and synovial fibroblast hyperplasia and induction of local glucocorticoid generation within synovium are needed for a significant paracrine effect of inflammation on bone. The data also suggest that in clinical situations where glucocorticoids are used to target synovial inflammation the detrimental effects of this therapy on bone could be mediated by direct effects through glucocorticoid receptor signalling in osteoblasts or indirectly through glucocorticoid receptor signalling in synovial fibroblasts.

The involvement of glucocorticoids in mechanisms to switch off tissue repair is not surprising. Glucocorticoids have anti-anabolic and catabolic effects in a range of tissues. An increase in the levels of glucocorticoids within the tissue through an increase in the activity of the hypothalamo-pituitary-adrenal axis is an important protective response. Adrenal insufficiency in humans or animals leads to a dramatic reduction in ability to withstand inflammatory stress [19]. Within bone, glucocorticoid signalling in synovial fibroblasts may provide a useful way of uncoupling bone resorption from formation during joint disease. Without an ability to temporarily uncouple formation from resorption there is a risk of aberrant, uncoordinated bone deposition that could be detrimental to the function of the joint. Importantly, abnormal osteophyte formation has been recently reported in HSD11B1 knockout mice in response to inflammatory arthritis [20]. At sites of bone remodelling, there was clearly abnormal excessive formation of new bone that was greatest adjacent to the site of synovial tissue inflammation. This is despite the gene for DKK1 being intact, and there being higher levels of circulating TNFα and endogenous corticosterone during inflammation, in this model. All these factors would normally be expected to result in a greater impairment of bone formation in knockout animals than wild types. The high corticosterone levels also demonstrate that the phenotype observed is unlikely to be related to an alteration of systemic glucocorticoid levels since excessive bone formation occurred despite the higher circulating glucocorticoid levels.

Previous studies have linked variation in the expression of DKK1 by synovial fibroblasts to rheumatic diseases associated with excessive bone formation, primarily AS [13,21] although abnormal expression of the osteocyte-specific protein (and Wnt signalling inhibitor) sclerostin has also been described [22]. We observed no difference in the ability of glucocorticoids to induce DKK1 in a limited number of patients with AS. However, it must be borne in mind that the excessive formation of bone in this condition is normally restricted to the axial spine. The reason for the axial predisposition to AS is unclear but it is possible that this reflects a difference in the regulation or expression of 11β-HSD1 in the spinal tissues. Synovial tissue is likely to differ between the peripheral and central joints and 11β-HSD1 expression in some cell types demonstrates regional variation [9]. Polymorphic markers within the HSD11B1 gene have been linked to differences in bone density and fracture risk [23] and could provide tools to examine for differences in bone manifestation of disease in patients with chronic inflammatory conditions.

## Conclusions

These data show that local glucocorticoid metabolism has an important role in the regulation of bone remodelling. The 11β-HSD1 enzyme is thus a potential therapeutic target for treating disorders characterised by uncoupling of bone formation from resorption.

## Abbreviations

AS: ankylosing spondylitis; DKK1: dickkopf-1; ELISA: enzyme-linked immunosorbent assay; GRα: glucocorticoid receptor alpha; 11β-HSD: 11beta-hydroxysteroid dehydrogenase; IL-1: interleukin 1; OA: osteoarthritis; PCR: polymerase chain reaction; RA: rheumatoid arthritis; TNFα: tumor necrosis factor alpha.

## Competing interests

The authors have no competing interests.

## Authors' contributions

RH, MJ, AN and JT performed DKK1 and molecular biology assays. EH supervised laboratory techniques and secured study funding. AF generated human primary synovial fibroblast cell lines and advised on study design. PMS, CDB, KR and MSC conceived of the study, coordinated its design, interpreted the data and secured study funding. All authors read and approved the final manuscript.

## Supplementary Material

Additional file 1 **Table S1**. Complete list of genes included in array: Complete list of genes included in array examining the impact of TNFα and glucocorticoid treatments on Wnts, Wnt inhibitors and Wnt-regulated genes. Shaded rows indicates genes where expression was significantly impacted on by either TNFα or dexamethasone (DEX). Array data have been submitted to the Gene Expression Omnibus (GEO) repository and given the designation GSE37520. Click here for file
