# Supplementary material for: Synovial DKK1 expression is regulated by local glucocorticoid metabolism in inflammatory arthritis
Source: Arthritis Res Ther. 2012 Oct 18;14(5):R226. doi: 10.1186/ar4065 (PMC3580537; doi:10.1186/ar4065)
Supplement: Additional file 1 — Table S1. Complete list of genes included in array: Complete list of genes included in array examining the impact of TNFα and glucocorticoid treatments on Wnts, Wnt inhibitors and Wnt-regulated genes. Shaded rows indicates genes where expression was significantly impacted on by either TNFα or dexamethasone (DEX). Array data have been submitted to the Gene Expression Omnibus (GEO) repository and given the designation GSE37520. [file ar4065-S1.PPT]

## Slide 1
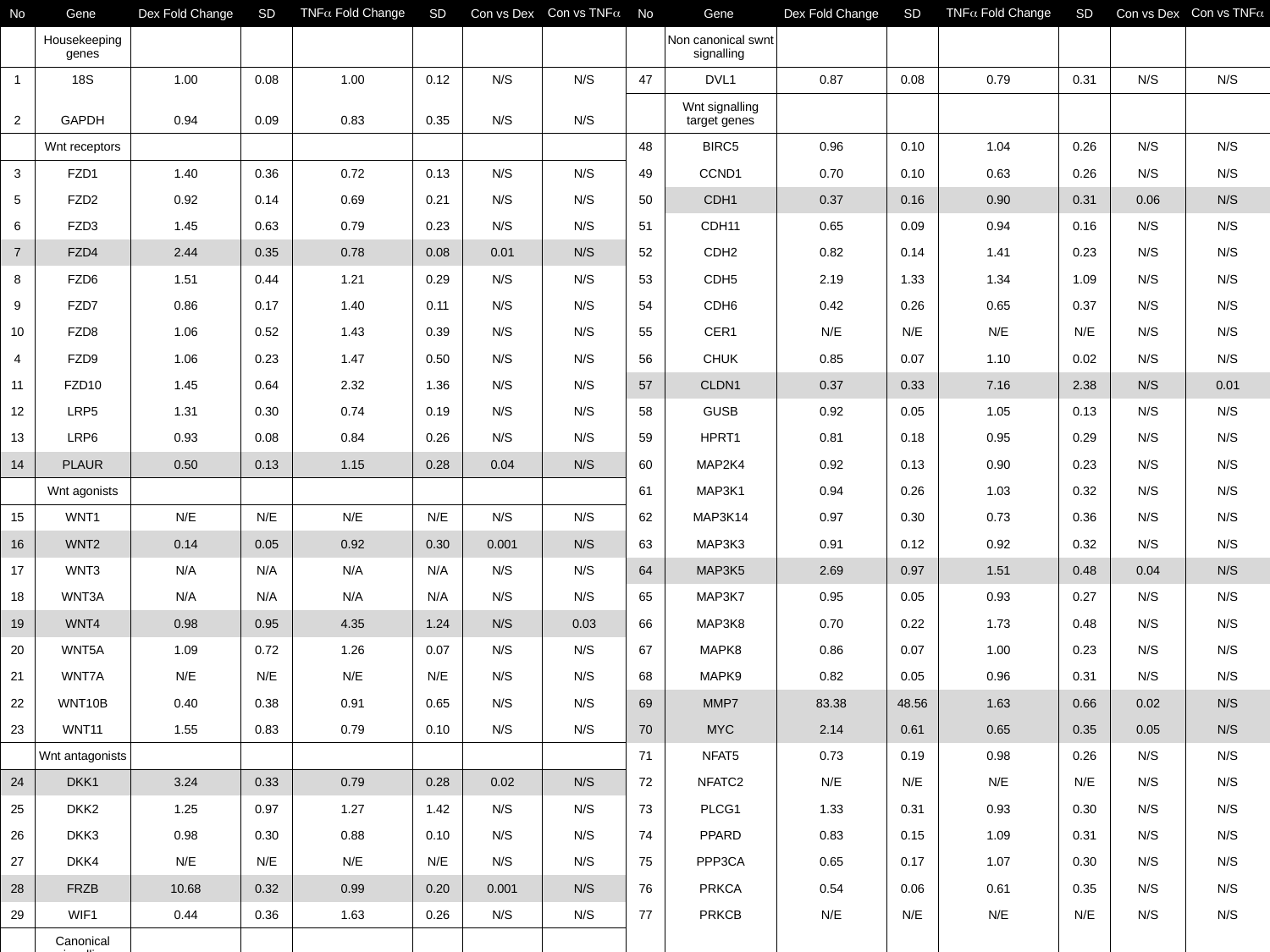

| No | Gene | Dex Fold Change | SD | TNFα Fold Change | SD | Con vs Dex | Con vs TNFα | No | Gene | Dex Fold Change | SD | TNFα Fold Change | SD | Con vs Dex | Con vs TNFα |
| --- | --- | --- | --- | --- | --- | --- | --- | --- | --- | --- | --- | --- | --- | --- | --- |
| | Housekeeping genes | | | | | | | | Non canonical swnt signalling | | | | | | |
| 1 | 18S | 1.00 | 0.08 | 1.00 | 0.12 | N/S | N/S | 47 | DVL1 | 0.87 | 0.08 | 0.79 | 0.31 | N/S | N/S |
| 2 | GAPDH | 0.94 | 0.09 | 0.83 | 0.35 | N/S | N/S | | Wnt signalling target genes | | | | | | |
| | Wnt receptors | | | | | | | 48 | BIRC5 | 0.96 | 0.10 | 1.04 | 0.26 | N/S | N/S |
| 3 | FZD1 | 1.40 | 0.36 | 0.72 | 0.13 | N/S | N/S | 49 | CCND1 | 0.70 | 0.10 | 0.63 | 0.26 | N/S | N/S |
| 5 | FZD2 | 0.92 | 0.14 | 0.69 | 0.21 | N/S | N/S | 50 | CDH1 | 0.37 | 0.16 | 0.90 | 0.31 | 0.06 | N/S |
| 6 | FZD3 | 1.45 | 0.63 | 0.79 | 0.23 | N/S | N/S | 51 | CDH11 | 0.65 | 0.09 | 0.94 | 0.16 | N/S | N/S |
| 7 | FZD4 | 2.44 | 0.35 | 0.78 | 0.08 | 0.01 | N/S | 52 | CDH2 | 0.82 | 0.14 | 1.41 | 0.23 | N/S | N/S |
| 8 | FZD6 | 1.51 | 0.44 | 1.21 | 0.29 | N/S | N/S | 53 | CDH5 | 2.19 | 1.33 | 1.34 | 1.09 | N/S | N/S |
| 9 | FZD7 | 0.86 | 0.17 | 1.40 | 0.11 | N/S | N/S | 54 | CDH6 | 0.42 | 0.26 | 0.65 | 0.37 | N/S | N/S |
| 10 | FZD8 | 1.06 | 0.52 | 1.43 | 0.39 | N/S | N/S | 55 | CER1 | N/E | N/E | N/E | N/E | N/S | N/S |
| 4 | FZD9 | 1.06 | 0.23 | 1.47 | 0.50 | N/S | N/S | 56 | CHUK | 0.85 | 0.07 | 1.10 | 0.02 | N/S | N/S |
| 11 | FZD10 | 1.45 | 0.64 | 2.32 | 1.36 | N/S | N/S | 57 | CLDN1 | 0.37 | 0.33 | 7.16 | 2.38 | N/S | 0.01 |
| 12 | LRP5 | 1.31 | 0.30 | 0.74 | 0.19 | N/S | N/S | 58 | GUSB | 0.92 | 0.05 | 1.05 | 0.13 | N/S | N/S |
| 13 | LRP6 | 0.93 | 0.08 | 0.84 | 0.26 | N/S | N/S | 59 | HPRT1 | 0.81 | 0.18 | 0.95 | 0.29 | N/S | N/S |
| 14 | PLAUR | 0.50 | 0.13 | 1.15 | 0.28 | 0.04 | N/S | 60 | MAP2K4 | 0.92 | 0.13 | 0.90 | 0.23 | N/S | N/S |
| | Wnt agonists | | | | | | | 61 | MAP3K1 | 0.94 | 0.26 | 1.03 | 0.32 | N/S | N/S |
| 15 | WNT1 | N/E | N/E | N/E | N/E | N/S | N/S | 62 | MAP3K14 | 0.97 | 0.30 | 0.73 | 0.36 | N/S | N/S |
| 16 | WNT2 | 0.14 | 0.05 | 0.92 | 0.30 | 0.001 | N/S | 63 | MAP3K3 | 0.91 | 0.12 | 0.92 | 0.32 | N/S | N/S |
| 17 | WNT3 | N/A | N/A | N/A | N/A | N/S | N/S | 64 | MAP3K5 | 2.69 | 0.97 | 1.51 | 0.48 | 0.04 | N/S |
| 18 | WNT3A | N/A | N/A | N/A | N/A | N/S | N/S | 65 | MAP3K7 | 0.95 | 0.05 | 0.93 | 0.27 | N/S | N/S |
| 19 | WNT4 | 0.98 | 0.95 | 4.35 | 1.24 | N/S | 0.03 | 66 | MAP3K8 | 0.70 | 0.22 | 1.73 | 0.48 | N/S | N/S |
| 20 | WNT5A | 1.09 | 0.72 | 1.26 | 0.07 | N/S | N/S | 67 | MAPK8 | 0.86 | 0.07 | 1.00 | 0.23 | N/S | N/S |
| 21 | WNT7A | N/E | N/E | N/E | N/E | N/S | N/S | 68 | MAPK9 | 0.82 | 0.05 | 0.96 | 0.31 | N/S | N/S |
| 22 | WNT10B | 0.40 | 0.38 | 0.91 | 0.65 | N/S | N/S | 69 | MMP7 | 83.38 | 48.56 | 1.63 | 0.66 | 0.02 | N/S |
| 23 | WNT11 | 1.55 | 0.83 | 0.79 | 0.10 | N/S | N/S | 70 | MYC | 2.14 | 0.61 | 0.65 | 0.35 | 0.05 | N/S |
| | Wnt antagonists | | | | | | | 71 | NFAT5 | 0.73 | 0.19 | 0.98 | 0.26 | N/S | N/S |
| 24 | DKK1 | 3.24 | 0.33 | 0.79 | 0.28 | 0.02 | N/S | 72 | NFATC2 | N/E | N/E | N/E | N/E | N/S | N/S |
| 25 | DKK2 | 1.25 | 0.97 | 1.27 | 1.42 | N/S | N/S | 73 | PLCG1 | 1.33 | 0.31 | 0.93 | 0.30 | N/S | N/S |
| 26 | DKK3 | 0.98 | 0.30 | 0.88 | 0.10 | N/S | N/S | 74 | PPARD | 0.83 | 0.15 | 1.09 | 0.31 | N/S | N/S |
| 27 | DKK4 | N/E | N/E | N/E | N/E | N/S | N/S | 75 | PPP3CA | 0.65 | 0.17 | 1.07 | 0.30 | N/S | N/S |
| 28 | FRZB | 10.68 | 0.32 | 0.99 | 0.20 | 0.001 | N/S | 76 | PRKCA | 0.54 | 0.06 | 0.61 | 0.35 | N/S | N/S |
| 29 | WIF1 | 0.44 | 0.36 | 1.63 | 0.26 | N/S | N/S | 77 | PRKCB | N/E | N/E | N/E | N/E | N/S | N/S |
| | Canonical signalling mediators | | | | | | | 78 | PRKCD | 1.05 | 0.22 | 1.07 | 0.26 | N/S | N/S |
| 30 | APC | 0.70 | 0.25 | 0.72 | 0.30 | N/S | N/S | 79 | PRKCE | 0.83 | 0.09 | 1.06 | 0.16 | N/S | N/S |
| 31 | AXIN1 | 0.95 | 0.04 | 1.04 | 0.29 | N/S | N/S | 80 | PRKCI | 0.89 | 0.07 | 0.85 | 0.38 | N/S | N/S |
| 32 | CTNNA1 | 0.88 | 0.08 | 0.89 | 0.12 | N/S | N/S | 81 | PRKCZ | 0.66 | 0.16 | 0.74 | 0.32 | 0.01 | N/S |
| 33 | CTNNB1 | 0.66 | 0.08 | 1.34 | 0.16 | N/S | N/S | 82 | PTGS2 | 0.61 | 0.32 | 8.20 | 1.78 | N/S | 0.05 |
| 34 | GSK3B | 0.80 | 0.20 | 0.82 | 0.23 | N/S | N/S | 83 | RAC1 | 0.86 | 0.18 | 0.85 | 0.30 | N/S | N/S |
| 35 | JUN | 0.46 | 0.21 | 0.81 | 0.19 | N/S | N/S | 84 | SMARCA4 | 0.91 | 0.21 | 0.99 | 0.28 | N/S | N/S |
| 36 | LEF1 | 1.80 | 0.49 | 2.49 | 0.74 | N/S | 0.04 | 85 | TFAM | 0.91 | 0.16 | 0.99 | 0.23 | N/S | N/S |
| 37 | TCF3 | 0.91 | 0.21 | 0.91 | 0.24 | N/S | N/S | 86 | TP53 | 0.93 | 0.21 | 1.02 | 0.40 | N/S | N/S |
| 38 | TCF4 | 0.87 | 0.19 | 1.01 | 0.07 | N/S | N/S | 87 | VIM | 0.58 | 0.43 | 0.96 | 0.27 | 0.01 | N/S |
| 39 | TCF7 | 0.38 | 0.19 | 0.78 | 0.45 | 0.02 | N/S | 88 | ZEB1 | 1.68 | 0.46 | 0.84 | 0.23 | N/S | N/S |
| 40 | TCF7L2 | 0.84 | 0.27 | 0.81 | 0.18 | N/S | N/S | | Transcription factors | | | | | | |
| | Degradation of beta catenin | | | | | | | 89 | CEBPB | 1.33 | 0.26 | 1.19 | 0.20 | N/S | N/S |
| 42 | PSMB10 | 0.89 | 0.07 | 1.98 | 0.45 | N/S | 0.04 | 90 | CREB1 | 0.85 | 0.07 | 0.91 | 0.16 | N/S | N/S |
| 43 | PSMB8 | 0.66 | 0.16 | 1.62 | 0.63 | N/S | N/S | 91 | CREBBP | 0.88 | 0.33 | 0.90 | 0.19 | N/S | N/S |
| 44 | PSMC4 | 0.88 | 0.08 | 1.31 | 0.21 | N/S | N/S | 92 | FOSL1 | 1.45 | 0.64 | 1.14 | 0.65 | N/S | N/S |
| 41 | UBB | 0.44 | 0.06 | 1.23 | 0.17 | 0.01 | N/S | 93 | HNF1A | N/E | N/E | N/E | N/E | N/S | N/S |
| 45 | UBC | 1.05 | 0.36 | 1.07 | 0.18 | N/S | N/S | 94 | HNF1B | N/E | N/E | N/E | N/E | N/S | N/S |
| 46 | UBD | 1.25 | 0.36 | 59.45 | 36.35 | N/S | 0.001 | 95 | HNF4A | N/E | N/E | N/E | N/E | N/S | N/S |
| | | | | | | | | 96 | REST | 0.84 | 0.27 | 0.97 | 0.30 | N/S | N/S |
